# Phylogenetic evidence for intratypic recombinant events in a novel human adenovirus C that causes severe acute respiratory infection in children

**DOI:** 10.1038/srep23014

**Published:** 2016-03-10

**Authors:** Yanqun Wang, Yamin Li, Roujian Lu, Yanjie Zhao, Zhengde Xie, Jun Shen, Wenjie Tan

**Affiliations:** 1 Key Laboratory of Medical Virology, Ministry of Health; National Institute for Viral Disease Control and Prevention, China CDC, Beijing 102206, China; 2 Key Laboratory of Major Diseases in Children and National Key Discipline of Pediatrics Capital Medical University, Ministry of Education, Beijing Pediatric Research Institute, Beijing Children’s Hospital, Capital Medical University, Beijing 100045, China; 3 Children Hospital of Fudan University, Shanghai 200032, China

## Abstract

Human adenoviruses (HAdVs) are prevalent in hospitalized children with severe acute respiratory infection (SARI). Here, we report a unique recombinant HAdV strain (CBJ113) isolated from a HAdV-positive child with SARI. The whole-genome sequence was determined using Sanger sequencing and high-throughput sequencing. A phylogenetic analysis of the complete genome indicated that the CBJ113 strain shares a common origin with HAdV-C2, HAdV-C6, HAdV-C1, HAdV-C5, and HAdV-C57 and formed a novel subclade on the same branch as other HAdV-C subtypes. BootScan and single nucleotide polymorphism analyses showed that the CBJ113 genome has an intra-subtype recombinant structure and comprises gene regions mainly originating from two circulating viral strains: HAdV-1 and HAdV-2. The parental penton base, pVI, and DBP genes of the recombinant strain clustered with the HAdV-1 prototype strain, and the E1B, hexon, fiber, and 100 K genes of the recombinant clustered within the HAdV-2 subtype, meanwhile the E4orf1 and DNA polymerase genes of the recombinant shared the greatest similarity with those of HAdV-5 and HAdV-6, respectively. All of these findings provide insight into our understanding of the dynamics of the complexity of the HAdV-C epidemic. More extensive studies should address the pathogenicity and clinical characteristics of the novel recombinant.

Human adenoviruses (HAdVs) are highly contagious pathogens with more than 60 serotypes which have been recognized and divided into seven species (A-G)^1^. At present, human adenovirus infection is mainly characterized by acute respiratory infections, gastrointestinal diseases, ophthalmic infection and genitourinary system illness^2^,^3^,^4^. Of the HAdV pathogens associated with respiratory disease, HAdV-B (HAdV-3, HAdV-7) and HAdV-C (HAdV-1, HAdV-2, HAdV-5 and HAdV-6) are among the most commonly reported and associated with severe acute respiratory infection (SARI)^5^,^6^.

The current classification of HAdV is based mainly on the marked nucleotide sequence divergence within the hexon and penton genes, which corresponds to serotype neutralisation^6^. The molecular typing by HAdV hexon sequences have been conducted in many countries to accelerate the discrimination of different types, resulting in timely improved patient care^5^.

However, novel strains always arise from mutations or intratypic recombination among different types of HAdV^7^,^8^. Recently, multiplex novel adenoviruses were detected in wild animals, such as the South Polar Skua (*Stercorarius maccormicki*)^9^, Chinstrap Penguin (*Pygoscelis antarctica*)^10^ and gull^11^. Recombinant adenoviruses have also been sporadically identified in human, especially for HAdV-D and HAdV-B^8^,^12^,^13^ species. However, few reports have been examined genetic recombinant of HAdV-C other than HAdV-C57^14^.

SARI is a serious threat to children and infants, who require hospitalization, and several reports have shown an association between SARI in children and specific species or novel recombinant strains of HAdV^5^. Here, we describe the detection and characterization of a novel subtype of HAdV-C (CBJ113 strain, accession number: KR699642) isolated from a hospitalized child with SARI. An epidemiological survey and genetic recombinant analyses were also performed to determine the prevalence and recombination pattern.

## Results

### Prevalence of a novel HAdV-C subtype in China

**Figure 1:**
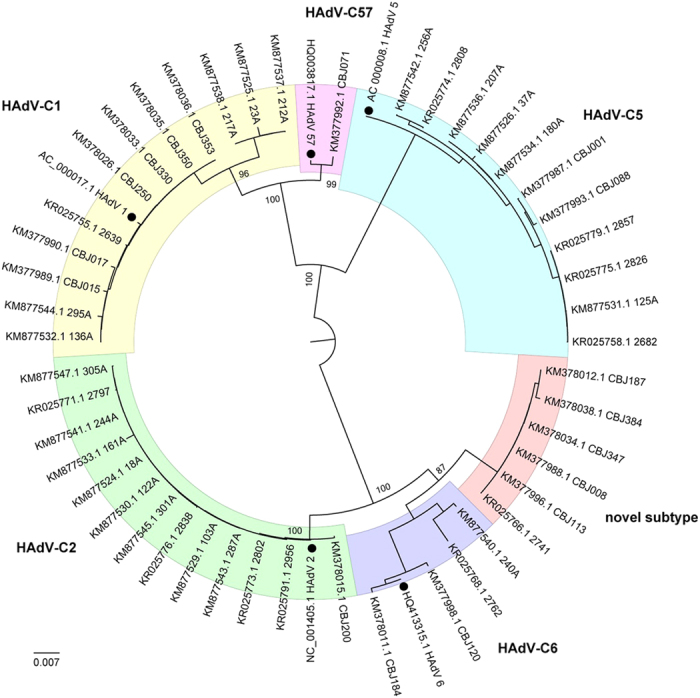
Phylogenetic relationships based on partial hexon gene sequences of all the HAdV-C strains detected in this study. All 47 HAdV-C strains detected in this study and five typical sequences (indicated with solid circles) of HAdV-C were analyzed using Neighbor-Joining method with 1000 bootstrap replicates implement in the MEGA 5.0 software. In addition to the five common genotypes (HAdV-1, HAdV-2, HAdV-5 HAdV-6 and HAdV-57), a novel subtype (6 strains) was detected in China.

To determine the prevalence of HAdV among children with SARI in China, all of the respiratory samples collected in this study were used for nested PCR analysis. Of the 993 respiratory specimens, 127 (12.8%) were positive for HAdV by PCR. Of these, six strains segregated as a novel HAdV-C subtype in a phylogenetic analysis of partial 3’-terminal sequence of hexon gene (Fig. 1). Of the six novel strains, five were from Beijing and one was from Shanghai. In total, the novel subtype accounted for 4.7% of all HAdV infections. The novel subtype may have been prevalent in China for a long time considering the collection period from 2007 to 2014.

### Isolation and complete genomic characterization of the novel HAdV-C subtype

**Figure 2:**
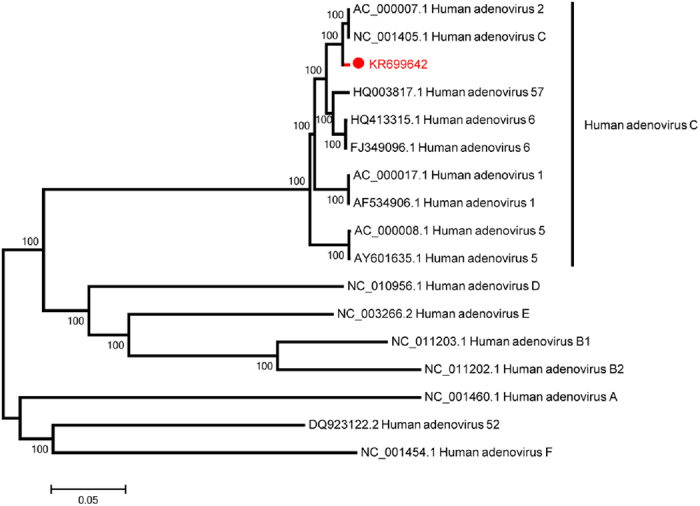
Phylogenetic analysis of HAdV based on the complete genomes. One novel HAdV-C subtype (indicated in red) and 12 other HAdV representative strains were analyzed using Neighbor-Joining method with 1000 bootstrap replicates in MEGA 5.0 program, number as the nodes represent bootstrap support.

Six specimens positive for the novel HAdV-C subtype were used initially for viral isolation. Of these, one isolated from a hospitalized pediatric patient caused a visible CPE on culturing. It was archived as strain CBJ113. Using next-generation and Sanger sequencing, the full-length genomic sequence of strain CBJ113 was determined, and the genomic data, noted formally as “human mastadenovirus C strain CBJ113” and in this report as “CBJ113”, was deposited in GenBank (accession number KR699642). The genome contains 35,926 bp with a GC content of 55.2%; the plus strand had an overall base composition of 23.16% A, 27.96% C, 27.25% G, and 21.63% T. Similar to the genomes of other HAdV-C strains, 36 coding sequences were identified. Phylogenetic analysis of 12 archived complete HAdV genomes showed that CBJ113 shares a common origin with HAdV-1, HAdV-2, HAdV-5, HAdV-6, and HAdV-57 (Fig. 2) and formed a new subclade on the same branch with other HAdV-C subtypes. Furthermore, the phylogenetic analysis of the corresponding partial 3′-terminal sequence of hexon gene yielded different topologies (Fig. 1), indicating potential genetic recombination events. Therefore, a novel HAdV-C subtype (CBJ113) circulating in China has been identified.

### Comparative genomic analysis

Based on the nucleotide alignment of the complete genomic sequence, the CBJ113 strain is conserved, sharing 99.22% nucleotide identity with the prototype strain of HAdV-2 (AC_000007) (Table 1). Comparison of the nucleotide sequences of the hexon and fiber genes showed the highest sequence similarity between strains CBJ113 and HAdV-2, with identities of 98.82~99.71%. On the other hand, the CBJ113 strain shared higher identities with a representative HAdV-1 (AC_000017.1) strain in the penton base (99.77%), pVI (99.87%), and DBP (98.92%) genes. HAdV-5 and HAdV-6 showed the greatest similarities to CBJ113 in the E4orf1 (98.43%) and DNA polymerase (99.86%) genes, respectively. Comparative genomics analysis indicated limited sequence variation in the novel HAdV-C type, and genetic recombination may play an important role in its origin and evolution.

### Genome recombination analysis

**Figure 3:**
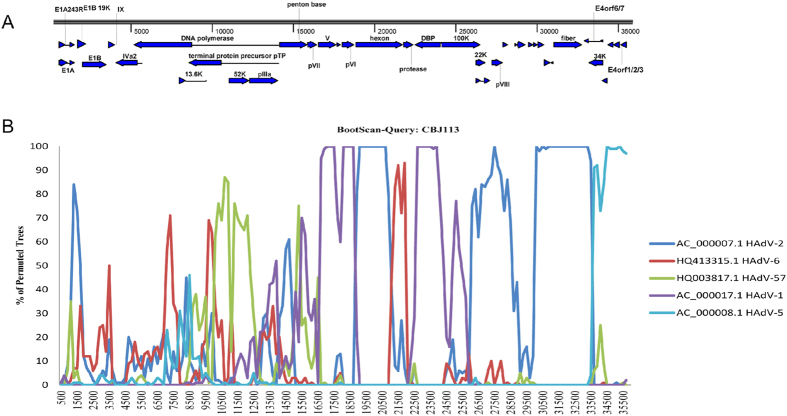
Genetic recombinant analyses of the complete genome of the novel HAdV-C subtype. (**A**) Genetic organization of HAdV-C. (**B**) BootScan analysis. CBJ113 was used as the query sequence and compared with other five representative strains of HAdV-C. The default setting of SimPlot software was used as follows: window size 1000bp, step size 200bp. 100 replicates used, gap stripping, distance model (Kimura) and tree model (neighbor-joining).

**Figure 4:**
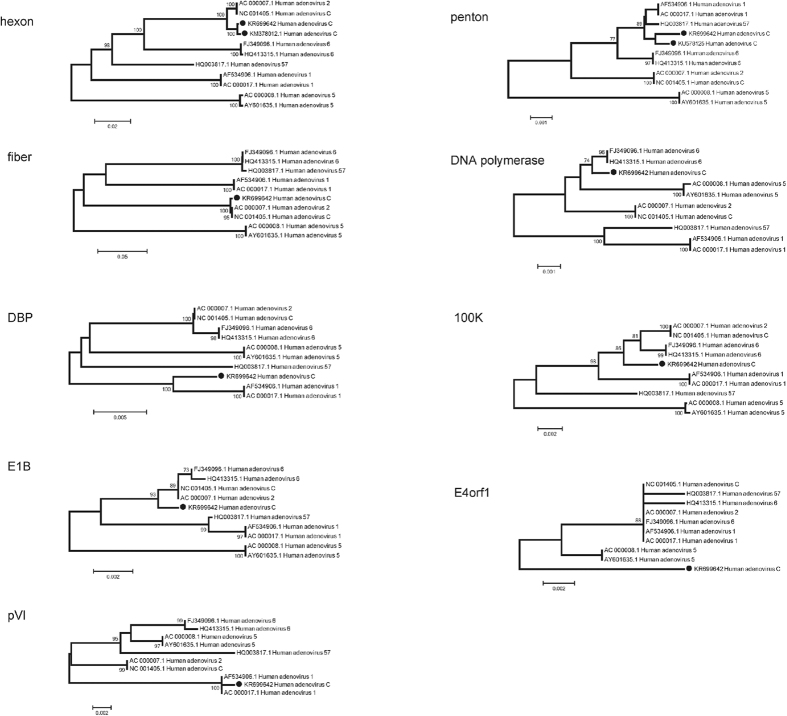
Phylogenetic analyses of several functional genes of HAdV-C strain CBJ113. The genes assessed in the phylogenetic analyses include fiber, hexon, penton, 100K, DBP, DNA polymerase, E1B, pVI and E4orf1. CBJ113 strain (KM377996.1), CBJ187 strain (KM378012.1 and KU578125) indicated by solid circles and other HAdV-C representative strains were analyzed by MEGA 5.0 software using Neighbor-joining method, with 1000 bootstrap replicates.

**Figure 5:**
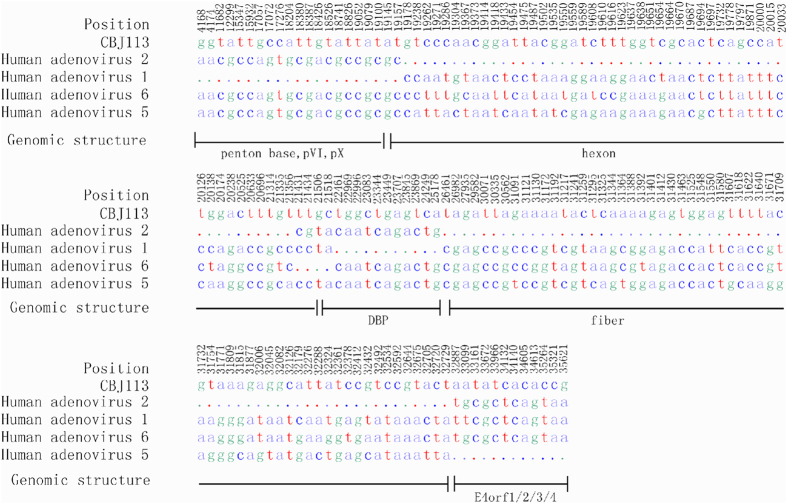
Single nucleotide difference between CBJ113 strain and consensus sequences of HAdV-1, HAdV-2, HAdV-5, HAdV-6 and HAdV-57. One hundred and fifty eight representative SNPs were summarized and used as special molecular markers for distinguishing different genotypes of HAdV-C. The same base in column was indicated dot.

To identify the occurrence of recombination events within the genome of CBJ113, we performed similarity plot and BootScan analyses using SimPlot software. BootScan analysis suggests the potential recombination events with signal of 80% or more of the observed permuted trees (Fig. 3). The results showed that CBJ113 is a recombinant harboring the penton base gene of HAdV-1 and the hexon and fiber genes of HAdV-2. In addition, partial sequence of DNA polymerase and E4orf1 may have derived from HAdV-6 and HAdV-5, separately. Furthermore, the sub-region phylogenetic trees analysis of CBJ113 sequence indicated a mosaic structure comprised of gene regions mainly originating from two circulating viral strain: HAdV-1 and HAdV-2 (Fig. 4). The parental penton base, pVI and DBP of the recombinant cluster with HAdV-1 prototype strain, and the E1B, hexon, fiber and 100 K genes of the recombinant cluster within the HAdV-2 subtype, although the DNA polymerase and E4orf1 genes of CBJ113 showed maximum homology with HAdV-6 and HAdV-5, respectively. Partial genome sequences of another stain CBJ187 were obtained and used for phylogenetic analysis, re-confirming the recombination event between HAdV-1 and HAdV-2 (Fig. 4). Single nucleotide polymorphisms (SNPs) have been used as molecular markers to distinguish different lineages in the typing of some viruses^15^,^16^. Here, we compared the SNPs of CBJ113 with the consensus sequences of HAdV-1, HAdV-2, HAdV-5 and HAdV-6. There are 158 representative SNPs presented in Fig. 5 which were lineage specific and could be used as markers to distinguish different subtypes. Within the nucleotide regions 4,168-19,157 and 22,461-25,178, the SNP pattern of CBJ113 was almost identical to that of HAdV-1. While within the nucleotide regions 19,178-21,356 and 26,461-32,729, the SNP pattern was identical to that of HAdV-2. The results coincided with BootScan and phylogenetic analyses. These findings indicated that CBJ113 arose from intratypic recombination events that have occurred among HAdV-C strains, including HAdV-1, and HAdV-2.

## Discussion

Human adenoviruses are ubiquitous in the environment and they are a frequent cause of respiratory infection or even life-threatening infection in children. Traditionally, adenoviruses are genotyped by PCR using sequence-specific primers. However, recombination was a hallmark of adenovirus genetics and frequent natural recombination has important implication for viral detection and pathogenicity. Here, we found a novel recombinant of HAdV-C (strain CBJ113), isolated in China. SNPs, BootScan and phylogenetic analyses all indicated that the novel subtype resulted from recombinant involving in HAdV-C1, HAdV-C2, HAdV-C5 and HAdV-C6 and provided evidence of intratypic recombination events in its evolutionary history. Although some previous studies have provided evidence for the mechanisms of recombination in HAdV-A 17, HAdV-B and HAdV-D 12,13,18, little research on HAdV-C recombinants has been reported, except HAdV-57^14^. Furthermore, the mosaic structure of the recombinant genome of CBJ113 was distinct from that of HAdV-57 or any other known HAdV-C, The only other mosaic recombinant used to be documented in HAdV-D^18^. It is difficult to confirm the time of genetic recombination; more sequence information was needed to explore the spatiotemporal relationships of the novel HAdV-C subtype in China.

In the present study, 127 hospitalized children with SARI were tested to be positive for HAdV. Of these, 6 strains belonged to the novel genotype of HAdV-C, accounting for 4.7% of all HAdV-positive cases. The emergence of the novel recombinant HAdV-C implied that these recombination events occur relatively frequently and the novel subtype of HAdV-C seems to be prevalent in China for a long period of time. More accurate epidemiological data are needed to investigate the HAdV population.

Co-infection is required to induce natural recombination, in China most HAdVs detected in SARI children belong to the species HAdV-B (HAdV-3 and HAdV-7) and HAdV-C (HAdV-1 and HAdV-2)^5^,^6^. Among the four HAdV species C, HAdV-1 and HAdV-2 were reported to cause a higher morbidity rate than HAdV-5 and HAdV-6. The circulating HAdV types in China were consistent with genetic recombinant type and showed an ongoing generation of recombinant strains among HAdV.

Regarding pathogenicity and tropism, the strain CBJ113 was isolated from a patient’s nasopharyngeal swab, and cultured in cell lines (HeLa and Hep2 cells) producing obvious CPE. Genomic analysis indicated that the penton base and fiber genes, which mediate attachment of adenovirus, were identical to those of HAdV-C. The tropism of the novel recombinant should be similar to that of other HAdV-C subtypes. Further researches are required to determine whether the emergence of recombination might affect its pathogenicity and virulence. It is not clear whether the novel HAdV-C subtype will maintain long-term stability.

In conclusion, the present study confirmed that a novel recombinant of HAdV-C circulated in China and molecular epidemiological surveillance suggested the novel recombinant have been prevalent in China for a long time. Further molecular surveillance of HAdV is necessary to explore its pathogenicity and clinical characteristics.

## Methods

### Sample collection and handling

During September 2007 to March 2014, 993 nasopharyngeal aspirates or induced sputum were collected from hospitalized children with SARI in Beijing (n = 259), Shanghai (n = 441) and Zhejiang Province (n = 293). Following the World Health Organization protocol^19^, all the respiratory samples were collected by medical professionals, and stored in −80 °C.

This study protocol was approved by the institutional ethics committee of Beijing Children’s Hospital, Children’s Hospital of Fudan University, Wenzhou Medical College, and Chinese Center for Disease Control and Prevention and was carried out in accordance with the approved guidelines. Written informed consent was obtained on their behalf from parents or guardian.

### Adenovirus identification and isolation

For molecular detection, nucleic acid was extracted from specimens using QIAamp MinElute Virus Spin Kit (QIAGEN, Germany) according to the manufacturer’s instruction. Partial 3’-terminal sequence of hexon gene was amplified by nested-PCR assay as previously described^20^,^21^. Adenovirus-positive samples were inoculated into two cell lines (HeLa and Hep2 cell) and grown in Dulbecco’s minimum essential medium (DMEM). Cytopathic effect (CPE) was monitored for at least 9 days after inoculation. Virus-infected cells were collected and used for subsequent detection and genome sequencing.

### Full-length genomic sequencing and annotation

After the pretreatment of infected cells, viral genomic DNA was used for random amplification as previously described^22^, the amplified DNA was used as a template for Illumina HiSeq2500 sequencing (paired-end, 2 × 125 bp), following the next-generation sequencing protocol of Beijing Berry Genomics. The complete genome of HAdV was assembled by SOAPdenovo^23^, SeqMan software and annotated based on the annotation of the HAdV-C prototype strain (NC_001405).

### Phylogenetic and recombinant analyses

Phylogenetic analyses were conducted using the Neighbor-Joining method using MEGA 5.0 24 with 1000 bootstrap replications. Bootstrap values greater than 70% were considered statistically significant for grouping. Bootscanning and Similarity plot analyses were performed using the default settings of SimPlot software^25^; the sequence of CBJ113 strain was used as the query sequence and compared to those of other HAdV-C subtypes with a sliding window of 1000 nucleotides. Single nucleotide difference analysis was used to confirm the possible recombinant events. The following archived HAdV genome sequences from GenBank were used for phylogenetic and genetic recombinant analysis, these are as follows: HAdV-A (NC_001460.1), HAdV-B1 (NC_011203.1), HAdV-B2 (NC_011202.1), HAdV-D (NC_010956.1), HAdV-E (NC_003266.2), HAdV-F (NC_001454.1), HAdV-G (DQ923122.2), HAdV-1 (AC_000017.1), HAdV-2 (AC_000007.1), HAdV-5 (AC_000008.1), HAdV-6 (HQ413315.1) and HAdV-57 (HQ003817.1).

### Nucleotide sequence accession number

All the complete or partial genome sequences were logged in the GenBank database under the following accession number: KM377987~KM378038, KM877524~KM877547, KR025750~KR025804, KU578125 and KP696777.

## Additional Information

**How to cite this article**: Wang, Y. *et al.* Phylogenetic evidence for intratypic recombinant events in a novel human adenovirus C that causes severe acute respiratory infection in children. *Sci. Rep.* **6**, 23014; doi: 10.1038/srep23014 (2016).

## Figures and Tables

**Table 1:** The nucleotide sequence identities between CBJ113 and HAdV-C representative strains.

Region	% nucleotide identity				
	CBJ113 strain				
	HAdV-C1	HAdV-C2	HAdV-C5	HAdV-C6	HAdV-C57
E1B	98.91%	**99.8%**	98.5%	99.66%	99.12%
DNA polymerase	98.76%	99.55%	99.38%	**99.86%**	98.87%
penton base	**99.77%**	99.24%	98.35%	99.53%	99.77%
pVI	**99.87%**	97.56%	97.29%	96.73%	96.3%
hexon	85.27%	**98.82%**	81.76%	89.55%	88.14%
DBP	**98.92%**	97.43%	96.84%	97.3%	96.97%
100K	98.87%	**99.38%**	97.34%	99.33%	98.02%
fiber	66.55%	**99.71%**	66.48%	70.05%	69.47%
E4orf1	98.16%	98.16%	**98.43%**	97.9%	97.89%
Complete genome	95.52%	**99.22%**	94.7%	97.35%	96.8%

The GenBank accession numbers of the representative strains are AC_000017 for HAdV-C1, AC_000007.1 for HAdV-C2, AC_000008 for HAdV-C5, HQ413315 for HAdV-C6 and HQ003817 for HAdV-C57. The highest similarity value in each row is in boldface.
